# Proximal congenital radial-ulnar synostosis and synchondrosis; pathogenic concept and a new therapeutic method

**Published:** 2013-12-25

**Authors:** G Burnei, RA Ghiță, AA Pârvan, E Japie, Ș Gavriliu, I Georgescu, T El Nayef, I Țiripa, Ș Hamei

**Affiliations:** * “Maria Sklodowska Curie" Emergency Hospital for Children, Bucharest; ** “Floreasca" Clinical Emergency Hospital, Bucharest; *** Tulcea County Hospital

**Keywords:** proximal radial-ulnar synostosis, forearm helical distortion, proximal radial-ulnar hemiarthroplasty, fibula-pro-radius, myo-osteo-arthroplastic reconstruction of the elbow

## Abstract

Abstract

Background context: Proximal congenital radial-ulnar synostosis (PCRUS) is defined by the development before birth of a bony bridge between the radius and ulna, usually at the proximal level, which blocks forearm rotation. This anomaly is rarely reported in the medical literature, because of its low prevalence, and treatment usually yields unsatisfactory results. The most commonly used surgical interventions are: forearm repositioning osteotomies with derotation of the radius and ulna, segmental resections of the middle third of the radius with muscular interposition, resection of the synostosis with the interposition of fatty tissue, tendons or fascia lata and resection of the proximal radius along with the transfer of the distal extensor carpi ulnaris tendon on the lateral edge of the radius.

Purpose: To describe a new treatment method for PCRUS, which we based on a new pathogenic concept, and to present our preliminary results.

Materials and method: Between 2011 and 2013 our team performed two myo-osteo-arthroplastic reconstructions of the elbow and forearm for PCRUS. The intervention involves the extraperiosteal stripping of the origins of the ventral forearm musculature, release of the interosseous membrane, resection of the proximal two thirds of the radius, reshaping of the synostosis, a double osteotomy of the ulna and the transfer and fixation of a proximal fibular graft, including the head with its articular cartilage, in place of the resected segment of the radius.

Results: Our preliminary study reveals favorable postoperative results, in comparison with other published methods. At the latest follow-up, one case had -10 degrees of pronation and 68 degrees of supination, and the other had 10 degrees of pronation and 66 degrees of supination.

Conclusions: Compared with other techniques, myo-osteo-arthroplastic reconstruction may seem overly invasive. However, the extent of this intervention is mandated by the pathogenic concepts of helical distortion, muscular retraction and anomalous configuration of the interosseous membrane. Benign cases do not require surgery. When there is no helical distortion, the intervention may be limited to the transfer of the proximal extremity of the fibula for the infant and small child.

## Pathogenic concept 

Within the upper limb bud, the elbow starts developing in the 34th day after fertilization, and the templates of the three bones that join at the elbow, the humerus, radius and ulna, appear on the 37th [**1**]. Because these cartilaginous templates begin as an unsegmented block, wrapped in a common perichondral membrane, a disrupting factor which acts during segmentation may cause the persistence of a bridge between the radius and ulna. The intensity and the interval in which these developmentally disruptive factors are present determine the extent of the synostosis and the position in which the forearm is fixed.

 During growth and development, the cartilaginous union becomes a bony bridge, as the templates of the radius and ulna ossify. The synchondrosis usually ossifies between 1 and 4 years old. 

 Among the 14 patients that our clinic treated, three were diagnosed right after birth. All these newborns diagnosed with PCRUS had their affected forearms fixed in supination, contrary to the established medical literature, which states that in PCRUS, forearms are always fixed in pronation [**1-3**]. This may be why very few newborns are diagnosed with this condition. Only a thorough physical examination may raise the suspicion of PCRUS, and a subsequent MRI examination establishes the diagnosis. During evolution, the extent of the union and the secondary alterations of forearm anatomy determine the degree of limitation to upper limb function.

 The alterations of the structures of the forearm progressively limit forearm rotation. At the age of 1, the motor deficit is not apparent or very slight. For most children with PCRUS, the limitation of pronation and supination is noticed by their parents between 1 and 3 years old, when the distortion of the radius and ulna lock the forearm in an intermediary or pronated position.

 Structurally, there are alterations of the shape, direction and configuration of the forearm bones, of the ventral muscles of the forearm and of the interosseous membrane.

 During growth, the three components of the malformation cause a helical distortion of the radius and ulna around the axis of the synostosis. The radial-carpal joint remains structurally and anatomically intact. 

**Fig. 1:**
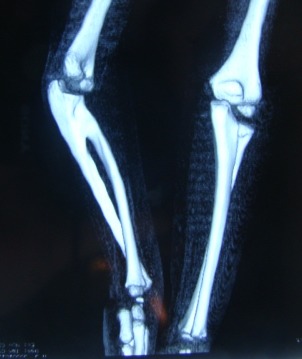
Helical distortion of the forearm bones (left side of image), compared to the opposite, normal, forearm

 A. Children with PCRUS and an extant radial head which only has the lateral-dorsal half of its growth plate also have a shortened radius relative to the ulna and frequently develop, in time, an accentuated helical configuration of the forearm bones (**Fig. 1**) and fixed pronation beyond 60 degrees, severely limiting hand function.

The extent of the helically distorted segment of the forearm corresponds to the position in which the hand is fixed. In severe forms, the distortion is maximum and the hand is fixed in extreme pronation. 

 B. The contracture and retraction of the ventral forearm muscles progresses along with the helical distortion. The function of the forearm muscles is also limited. These alterations require that the procedure includes the extraperiosteal stripping of the origins of the muscles responsible with pronation and supination. 

 C. The interosseous membrane is altered along with the bones it attaches to, which means that it must also be divided on its ulnar insertion.

## Materials and methods

Surgical technique The objective of this intervention is the reconstruction of the forearm bones, proximal joint and musculature (myo-osteo-arthroplastic reconstruction). The procedure was used on two patients at our clinic. The operation involves the following steps: 

 1. Extraperiostic stripping of the origins of the ventral forearm muscles and release of the interosseous membrane. 

 The incision starts on the medial fascial septum of the arm 4 cm above the medial epicondyle, continues along the ulnar septum of the forearm and ends at the ulnar styloid. The ulnar nerve is identified in its groove behind the medial epicondyle and is isolated to avoid it being injured. The fascia of the ventral forearm compartment is incised and the muscular insertions are cut above the periosteum from the radius, ulna and the medial epicondyle. Care must be taken not to injure the main vessels running ventral to the elbow, the median nerve and the anterior branch of the interosseous nerve, which is found on the ventral surface of the interosseous membrane. 

 The interosseous membrane is released by incising it on its ulnar border. 

 2. The resection of the proximal two thirds of the radius and the reshaping of the synostosis by abrasion. 

 The synostosis is cut longitudinally. If forearm supination is attempted at this stage, the helical distortion of the radius would not allow the physiological positioning of its distal end, thus requiring the partial resection of the radius in order to prevent recurrence. 

 The resection is meant to remove the distorted segment; usually, the lower limit of the resection is established by placing the hand in supination, so that the ventral face of the radius is placed in the anatomical position. The resection of the synostosis is finalized and the proximal ulna is shaped by abrasion in order for it to receive the prosthetic component for the proximal radial-ulnar hemiarthroplasty. 

 3. Double ulnar osteotomy 

 After stripping the periosteum and the removal of the proximal two thirds of the radius, the ulna is fully exposed and two osteotomies are performed: one 2 cm distal to the lower edge of the sigmoid notch and the other where the middle and distal third of the ulna meet. 

 The proximal osteotomy is mandated by the extension of the ulnar distortion to the olecranon: without it, the humeral-ulnar joint may become incongruent or dislocated when correction is attempted. The olecranon is repositioned and the osteotomy is fixed by using the ulnar prosthetic component, which has two screw holes, one proximal and one distal. The prosthesis has a double role, as a replacement joint surface and as an osteosynthesis device. 

 The second ulnar osteotomy is indicated if the distortion of the ulna extends beyond the proximal two thirds. When two osteotomies are performed, we recommend that the fragments are first aligned by using a thin elastic rod driven through the tip of the olecranon. The first osteotomy to be fixed should be the proximal one, using the prosthesis and two screws, and then the distal one using a half-cylinder plate with two screws. 

 4. Proximal fibula transfer 

 A proximal fibula graft, which includes the epiphysis, growth plate, articular surface and an adequate length of shaft to replace the resected radius, is harvested by using a classic approach, taking care to identify and isolate the fibular nerve.

 5. Checking the distal radial resection limit 

 Neglecting to perform this check may result in the persistence of a section of distorted radius, which may impede forearm rotation later on. If there remains a portion of distorted radius, the resection is repeated. 

 6. Fixation of the fibular graft 

**Fig. 2:**
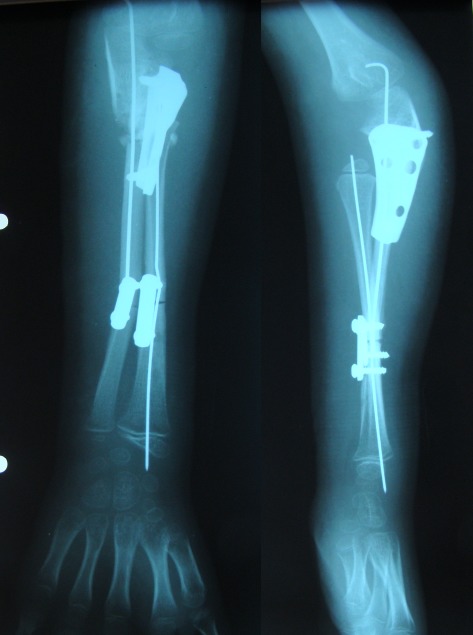
The final configuration of the forearm (postoperative radiograph). The fibular graft is fixed by using a plate with two screws and the two ulnar osteotomies are stabilized by the prosthesis and a plate with screws, respectively. Both bones are further stabilized by using a central pin or thin elastic rod

 The harvested fibular graft is aligned to the distal radius with the help of a thin elastic rod inserted through the distal radial metaphysis and is fixed using a half-cylinder plate and two screws. The final configuration of the forearm is shown in Fig. 2.

The manner of internal fixation presented above does not require immobilization and allows an active motion to be resumed at three weeks after surgery.

## Discussion

**Fig. 3:**
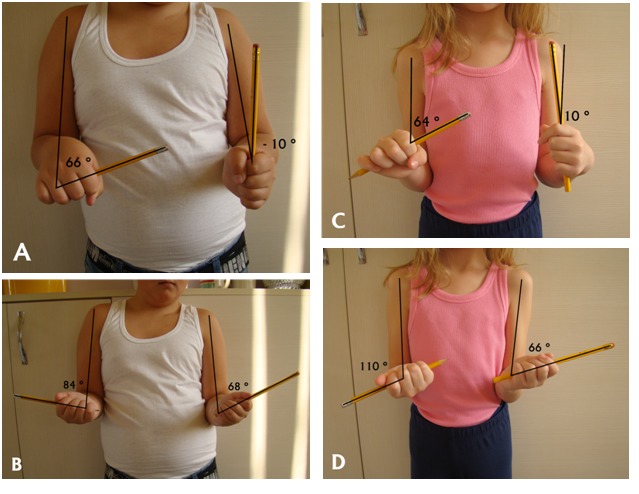
Clinical results for the two patients who underwent myo-osteo-arthroplastic reconstruction of the forearm. A, B: The male patient at almost 2 years follow-up (left forearm); C, D: The female patient at 9 months follow-up (left forearm). (A, C: maximum pronation; B, D: maximum supination)

Myo-osteo-arthroplastic reconstruction of the elbow joint is a laborious procedure, but, among other methods presented in the medical literature, it is the only one that achieved, at the latest follow-up (9 months and 2 years, respectively), an average of 67 degrees of forearm rotation. The two patients whom we operated by using this method, between 2011 and 2013, had good outcomes (**Fig. 3**), the patients and families are satisfied and now both children are involved in demanding activities (playing the piano and ballet, respectively).

In the 20 years leading up to 2011, the surgeons at the “Maria Sklodowska Curie" Emergency Hospital for Children in Bucharest operated proximal radial-ulnar synostoses by using a variety of procedures: repositioning osteotomies of the radius and ulna, segmental resection of the middle third of the radius with muscle interposition, resection of the synostosis with the interposition of fatty tissue, fascia lata or tendons and resection of the proximal radius along with the transfer of the insertion of the extensor carpi ulnaris to the lateral edge of the radius. 

 The repositioning osteotomies of the forearm are the most preferred by surgeons [**2**], due to the lower likelihood of failure. The most frequently used procedure is a transversal osteotomy through the synostosis [**2,4**], with some authors advocating for 5 mm of shortening. The forearm bones may be cut individually, distal to the synostosis, for example, in the middle of the ulna and through the distal radial metaphysis [**5**].

 For bilateral synostosis, Green and Mittal [**4**] recommend that the non-dominant forearm is placed in 20–35 degrees of supination and that the dominant one should be left as is, with the exception of cases with extreme pronation. Simmons [**6**] recommends 20 degrees of pronation for unilateral cases and, for bilateral involvement, 10–15 degrees of pronation for the dominant limb and neutral rotation for the non-dominant one.

 The restoration of forearm rotation can be attempted by using two types of procedures: on the one hand the resection of the synostosis and the interposition of a biological structure (fascia [**6**], muscle [**3,7**], fat [**8**], etc.) or an artificial material, and on the other hand the wide resection of the proximal radius, which allows the distal segment to rotate around the ulna [**9**]. The long-term outcomes of these procedures have yet to be determined [**2**] and published results are not encouraging given the frequent recurrences of the synostosis [**3**]. 

 The correction of the position of the forearm carries an important risk of complications because of soft tissue retraction, such as loss of correction, nervous or vascular injury and Volkmann contracture [**2**].

 In the 20 years before the development of this intervention, all the other procedures we used for PCRUS never yielded satisfactory results for either patients or parents. For this reason, the adoption of myo-osteo-arthroplastic reconstruction is an important step forward for those children with PCRUS who are involved in demanding activities, who need to have forearm rotation restored.

## Conclusions

Compared with other techniques, myo-osteo-arthroplastic reconstruction may seem overly invasive. However, the pathogenic concept of helical distortion, muscular retraction and abnormal interosseous membrane mandates its complexity.

 Cases with a benign evolution do not require surgery.

 For the newborn, infant and small child, the intervention may be limited to the transfer of the proximal fibula, but only if there is no helical distortion.
